# Improvement with Infliximab of a Disseminated Sarcoidosis in a Patient with Crohn's Disease

**DOI:** 10.1155/2014/368780

**Published:** 2014-02-06

**Authors:** Nader Chebib, Fabrice Piégay, Julie Traclet, François Mion, Jean-François Mornex

**Affiliations:** ^1^Hospices Civils de Lyon, 69003 Lyon, France; ^2^Université de Lyon, 69007 Lyon, France; ^3^Université Lyon1, 69007 Lyon, France; ^4^INRA, UMR754, 69007 Lyon, France

## Abstract

Sarcoidosis and Crohn's disease are systemic granulomatous disorders affecting the lung and the intestine, respectively, with variable involvement of other organs and are seldom associated. While anti-TNF**α** is a recognized treatment of Crohn's disease, its usage is discussed in sarcoidosis. A 42-year-old man presented with an 11-year-long history of Crohn's disease; upon discovery of an abnormal chest CT scan the diagnosis of multivisceral sarcoidosis was made and, later, a treatment with an anti-TNF**α** agent, infliximab, was started, because of worsening Crohn's disease recurrences. CT scan demonstrated net regression of pulmonary opacities and hepatosplenic lesions. Pathologies obtained from the intestinal tract and the bronchi of the patient were, respectively, characteristic of Crohn's disease and sarcoidosis leading to the diagnosis of both diseases. We report a rare case of steroid resistant Crohn's disease associated with multivisceral sarcoidosis, treated successfully by an anti-TNF**α** agent, infliximab.

## 1. Introduction

Both sarcoidosis and Crohn's disease are relapsing chronic inflammatory disorders characterized by the formation of granulomas; they share some organ locations, cytokine pathways, and genetic background. The association of both diseases has rarely been reported. If anti-TNF*α* is a recognized treatment of Crohn's disease, its role in sarcoidosis remains discussed. We report the association of both diseases in a single patient and the beneficial effect of anti-TNF*α* on both diseases.

## 2. Case Report

**Figure 1:**
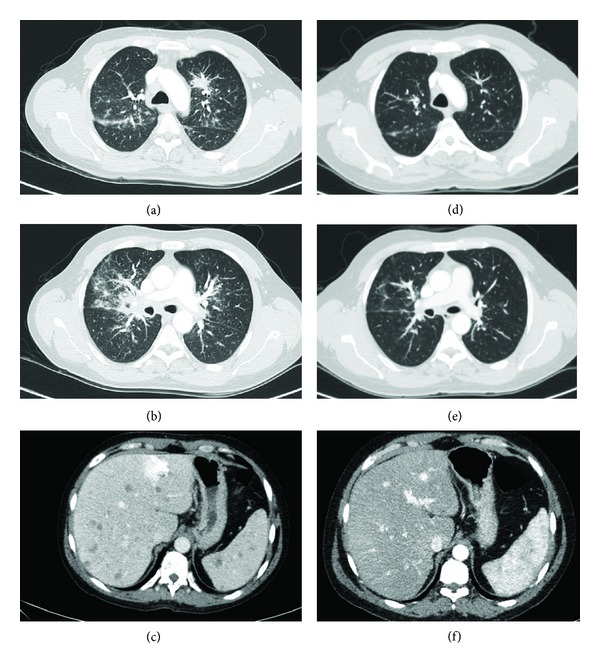
Chest ((a), (b), (d), and (e)) and abdominal ((c), (f)) CT scans showing resolution after treatment ((d), (e), and (f)) of diffuse lesions ((a), (b), and (c)).

**Figure 2:**
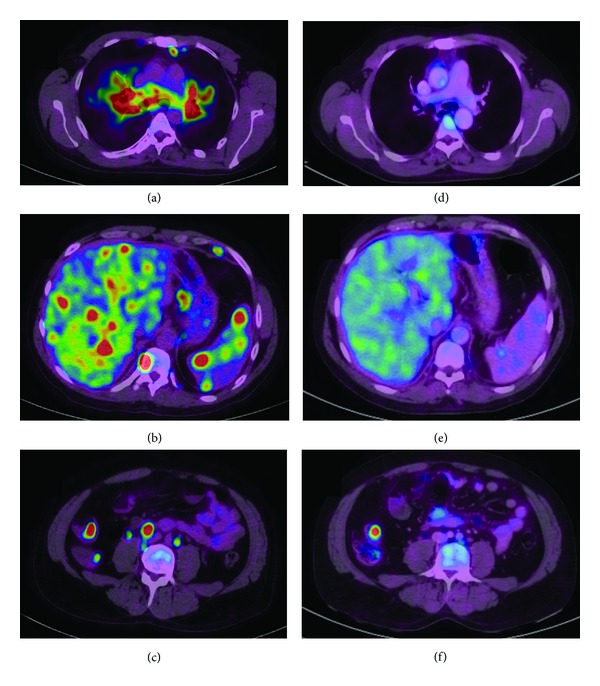
Chest ((a), (d)) and abdominal ((b), (c), (e), and (f)) PET scan showing hypermetabolism in pulmonary, hepatosplenic, spinal, and intestinal lesions before treatment ((a), (b), and (c)), markedly decreasing after treatment ((d), (e), and (f)) except for the distal ileum.

A 42-year-old man presented with a 3-month history of respiratory symptoms combining mild dyspnea, cough, asthenia, and weight loss. His past medical history included a 6-pack-year smoking habit and an 11-year-long history of Crohn's disease, treated with oral steroids (prednisolone 20 mg/day) ever since. Over time, repeated biopsies showing ulceration and transmural inflammatory lesions of the ileum, the colon, and the rectum including submucosal aggregates of lymphocytes and plasmocytes and, once, rectal epithelioid granulomas were always consistent with the diagnosis. Physical exam showed no abnormalities. Peripheral blood lymphocyte count was decreased (890/*μ*L including 374 CD4 lymphocytes/*μ*L, i.e., 42%); serum angiotensin converting-enzyme level was elevated (118 U/L). CT scan was markedly abnormal (Figures 1(a), 1(b), and 1(c)) showing enlarged bilateral hilar and mediastinal lymph nodes, a left apical irregular nodule, bilateral perihilar infiltrates, upper lobe micronodules, proximal bronchial thickening, and hepatic and splenic nodules, along with retroperitoneal lymph nodes. All of these lesions were metabolically active on PET scan as well as osteolytic spinal, costal, clavicular, and humeral lesions (Figures 2(a), 2(b), and 2(c)). Bronchoscopy showed a bilateral diffuse inflammatory infiltration, and the bronchoalveolar lavage disclosed a predominant neutrophilic alveolitis (77% neutrophils, 8% lymphocytes, CD4/CD8 lymphocyte ratio = 1.9) in the absence of any viral, bacterial, or fungal microorganism. Cultures were negative for *Mycobacterium *sp. Bronchial biopsies noted the presence of noncaseating granulomas with epithelioid and multinucleated giant cells. The diagnosis of multivisceral sarcoidosis was made and inhaled steroids to ease the cough were the only treatment instaured since the patient was already on oral steroids and the functional impact of the disease was mild; PFTs disclosed a mixed restrictive and obstructive pattern (Table 1). Four months later, a treatment with an anti-TNF*α* agent, infliximab, was started, because of worsening Crohn's disease recurrences; the dose regimen was 5 mg/Kg every 6 weeks. After 5 courses, oral steroids were successfully weaned, digestive symptoms were largely improved, the patient had gained weight and had no more cough, the dyspnea level diminished, and PFT abnormalities improved (Table 1). CT scan demonstrated net regression of pulmonary opacities and hepatosplenic lesions (Figures 1(d), 1(e), and 1(f)) with PET scan showing no more metabolic activity, except in the distal ileum area (SUV max 7.4) (Figures 2(d), 2(e), and 2(f)). Oral steroids were successfully weaned over a period of 6.5 months since the start of infliximab, and the patient remains off steroids to this day. Blood CD4 lymphocyte count increased slightly to 411/*μ*L (44%) and ACE level was not reassessed.

## 3. Discussion

Given the clinical presentation, the nosology of the digestive and the thoracic locations needs to be discussed. Gastrointestinal sarcoidosis, apart from being very rare, is mostly located in the stomach [1]. Lung involvement of inflammatory bowel diseases is mostly interstitial lung or bronchiolar disease and rarely granulomatous [2]. Lung nodules have been reported but are usually preceding Crohn's disease, mostly in children [3]. Finally, pathology obtained from the intestinal tract and the bronchi of the patient were, respectively, characteristic of Crohn's disease and sarcoidosis leading to the diagnosis of both diseases associated [4]. Of note, in a personal series, Reynolds reported 2 of 67 sarcoidosis patients with Crohn's disease [5]. Granulomatous-lymphocytic interstitial lung disease in common variable immunodeficiency [6] could be discussed. This patient presented, in addition to Crohn's disease, with a severe multiorgan sarcoidosis [5]. BAL in our patient showed neutrophilia, which is an uncommon feature in sarcoidosis, notably in the absence of an infectious disease or active smoking but it can be observed in chronic forms of sarcoidosis and stage IV disease [7]. The residual hypermetabolism in the ileum on the PET scan is consistent with a persistent mucosal inflammation due to Crohn's disease. Recently, genomic studies revealed common genetic variants predisposing to either disease: on one hand, different SNPs in the NOD2/CARD15 genes with relevance to the pathogenesis [8, 9] and on the other hand, single SNPs increasing the risk of either disease but with no mechanistic relevance so far [10, 11]. Increased local secretion has long suggested that TNF*α* among other cytokines may play a role in both diseases [12, 13]. While anti-TNF*α* is a recognized treatment of Crohn's disease [14], it has been reported to be of clinical use in refractory or severe sarcoidosis [15]. Sarcoidosis may occur during anti-TNF*α* treatment of Crohn's disease [16] and other inflammatory and rheumatic diseases [17, 18] and with other drugs interfering with the cell interaction processes [19]. In the present case, anti-TNF*α* was started after the diagnosis of sarcoidosis.

## 4. Conclusion

We report a rare case of steroid resistant Crohn's disease followed by multivisceral sarcoidosis, treated successfully by an anti-TNF*α* agent, infliximab. This is the first report to our knowledge of a simultaneous double response in both of these diseases to anti-TNF*α* therapy. Sarcoidosis and Crohn's disease are systemic granulomatous disorders affecting the lung and the intestine, respectively, with variable involvement of other organs. Differential is critical because, although they share many clinical, immunological, genetic and pathological aspects, treatment strategies are different including different clinical responses to anti-TNF*α*.

## Figures and Tables

**Table 1:** PFT results before and after treatment with infliximab.

	Before treatment	After treatment
FEV1/CV	63%	71%
FEV1	1.32 L (41%)	1.63 L (51%)
VC	2.08 L (52%)	2.31 L (59%)
RV	1.72 L (96%)	1.07 L (59%)
TLC	3.80 L (66%)	2.95 L (51%)
DLCO	35%	34%
KCO	79%	90%
